# The long non-coding RNA TUG1-miR-9a-5p axis contributes to ischemic injuries by promoting cardiomyocyte apoptosis via targeting KLF5

**DOI:** 10.1038/s41419-019-2138-4

**Published:** 2019-12-02

**Authors:** Di Yang, Jie Yu, Hui-Bin Liu, Xiu-Qing Yan, Juan Hu, Yang Yu, Jing Guo, Ye Yuan, Zhi-Min Du

**Affiliations:** 1 Institute of Clinical Pharmacy, the Second Affiliated Hospital of Harbin Medical University (The University Key Laboratory of Drug Research, Heilongjiang Province), Harbin, 150086 China; 2 Department of Clinical Pharmarcology (State-Province Key Laboratories of Biomedicine-Pharmaceutics of China, Key Laboratory of Cardiovascular Research, Ministry of Education), College of Pharmacy, Harbin Medical University, Harbin, 150086 China; 3 State Key Laboratory of Quality Research in Chinese Medicines, Macau University of Science and Technology, Macau, PR China

**Keywords:** Long non-coding RNAs, Cardiovascular diseases

## Abstract

Non-coding RNAs participate in many cardiac pathophysiological processes, including myocardial infarction (MI). Here we showed the interplay between long non-coding RNA taurine-upregulated gene 1 (lncR-TUG1), miR-9a-5p (miR-9) and Krüppel-like factor 5 (KLF5). LncR-TUG1 was upregulated in ischemic heart and in cultured cardiomyocytes exposed to H_2_O_2_. Knockdown of lncR-TUG1 markedly ameliorated impaired cardiac function of MI mice. Further study showed that lncR-TUG1 acted as a competitive endogenous RNA of miR-9, and silencing of lncR-TUG1 inhibited cardiomyocyte apoptosis by upregulating miR-9 expression. Furthermore, the miR-9 overexpression obviously prevented ischemia injury and significantly inhibited H_2_O_2_-induced cardiomyocyte apoptosis via inhibition of mitochondrial apoptotic pathway. KLF5, as a target gene of miR-9 by dual-luciferase reporter assay, was involved in the process of miR-9 in regulating cardiomyocyte apoptosis. Our data identified the KLF5 was downregulated by miR-9 overexpression and knockdown of KLF5 inhibited cardiomyocyte apoptosis induced by H_2_O_2_. MiR-9 exerts anti-cardiomyocyte apoptotic affects by targeting KLF5. Collectively, our data identify a novel function of lncR-TUG1/miR-9/KLF5 axis in regulating cardiomyocyte apoptosis that affects myocardial infarction progression.

## Introduction

Acute myocardial infarction (AMI), caused by the sudden occlusion of coronary flow, is one of the leading causes of morbidity and mortality worldwide. The prominent pathological change post-MI is death of cardiomyocytes, which can lead to the irreversible loss of heart function. Numerous studies have suggested that cardiomyocyte in the peri-infarct region may mostly undergo apoptosis, which can be potentially recovered from injury within a certain period of time post-MI if effective therapy was applied^1,2^. Therefore, excavating methods to inhibit cardiomyocyte apoptosis during early stage of MI might shed new light on the machinery that underlies ischemic heart disease regulation.

Long non-coding RNAs (lncRNAs) are a subfamily of RNAs longer than 200nt. LncRNAs participate in various cellular processes, including RNA processing^3^, structural scaffolds^4^, chromatin modification^5^, modulation of apoptosis and invasion^6^. Also, lncRNAs have been identified in cardiomyocytes and found to be essential for the development and progression of heart diseases^7,8^. Taurine-upregulated gene 1 (TUG1), located at chromosome 22q12, has been shown to play critical roles in multiple biologic processes^9^. The dysregulation of lncR-TUG1 participated in the development of several cancers^10,11^ and was related to the pathogenesis of many nervous system diseases^12,13^. Overexpression of lncR-TUG1 has recently been found to be involved in cell apoptosis such as endothelial cell in atherosclerosis^14^, in human glioma^13^ and in ischemic stroke^15^. These studies further substantiate the notion that lncR-TUG1 plays an improtant role in cell apoptosis. However, the study of lncR-TUG1 in cardiac diseases has been sparse and the functional role of lncR-TUG1 in MI remains to be elucidated.

MicroRNAs (miRNAs) are a class of small non-coding RNA molecules (containing 19–22 nucleotides) that are critical to a wide variety of biological processes^16,17^. MiRNAs act in modulation of multiple genes and subsequent downstream gene networks to affect cell growth, proliferation, differentiation and survival^18–20^. Recently, increasing evidence has implicated a regulatory mechanism by which lncRNAs function as a ceRNA to sponge endogenous miRNAs via interfering with miRNAs. It has been shown that lncR-TUG1 could directly bind to miR-9 by RNA pull-down assay^15^ and dual-luciferase reporter assay^21^. MiR-9 is a highly conserved mature miRNA across species and previous studies revealed the progressive role in cancer^22–24^. Recent evidence indicates that miR-9 promotes cell proliferation and inhibits apoptosis^25,26^ and may also regulate cardiomyocyte growth in response to cardiac hypertrophy via regulation of myocardin expression^27^. In addition, miR-9 was verified to inhibit hyperglycemia-induced pyroptosis in human ventricular cardiomyocyte^28^. However, the potential role of miR-9 in regulation of MI remains unclear. Therefore, we speculated that regulation of miR-9 by lncR-TUG1 may be involved in the ischemia injury-induced apoptosis.

KLF5 (Krüppel-like zinc-finger transcription factor 5), a member of the Krüppel-like transcription factor family, also known as BTEB2 and IKLF, which has diverse functions during cell differentiation and embryonic developments. We speculated that KLF5 might be the target of miR-9. KLF5 has been implicated as a tumor suppressor in breast, prostate, and intestinal cancers^29–31^, regulate fundamental cellular responses such as growth, apoptosis, angiogenesis, and proliferation^32–34^. KLF5 is a crucial determinant of the cellular response to cardiovascular injury, playing a key role in mediating tissue remodeling and pressure overload-mediated cardiac hypertrophy^35^.

Our present work reveals that lncR-TUG1 is involved in the regulation of cardiomyocyte apoptosis post-MI. LncR-TUG1 as an endogenous sponge that competitively binds miR-9. Silencing of lncR-TUG1 inhibits cardiomyocyte apoptosis in vivo and in vitro by upregulating miR-9. We further find that KLF5 is a target of miR-9 and miR-9 participates in the regulation of KLF5 expression. In short, lncR-TUG1 regulates cardiomyocyte apoptosis through targeting the miR-9/KLF5 pathway. Our data shed new light on the understanding of interaction between lncRNA and miRNA in molecular regulation of ischemic heart injuries.

## Results

### LncR-TUG1 as a regulator of myocardial infarction

**Fig. 1:**
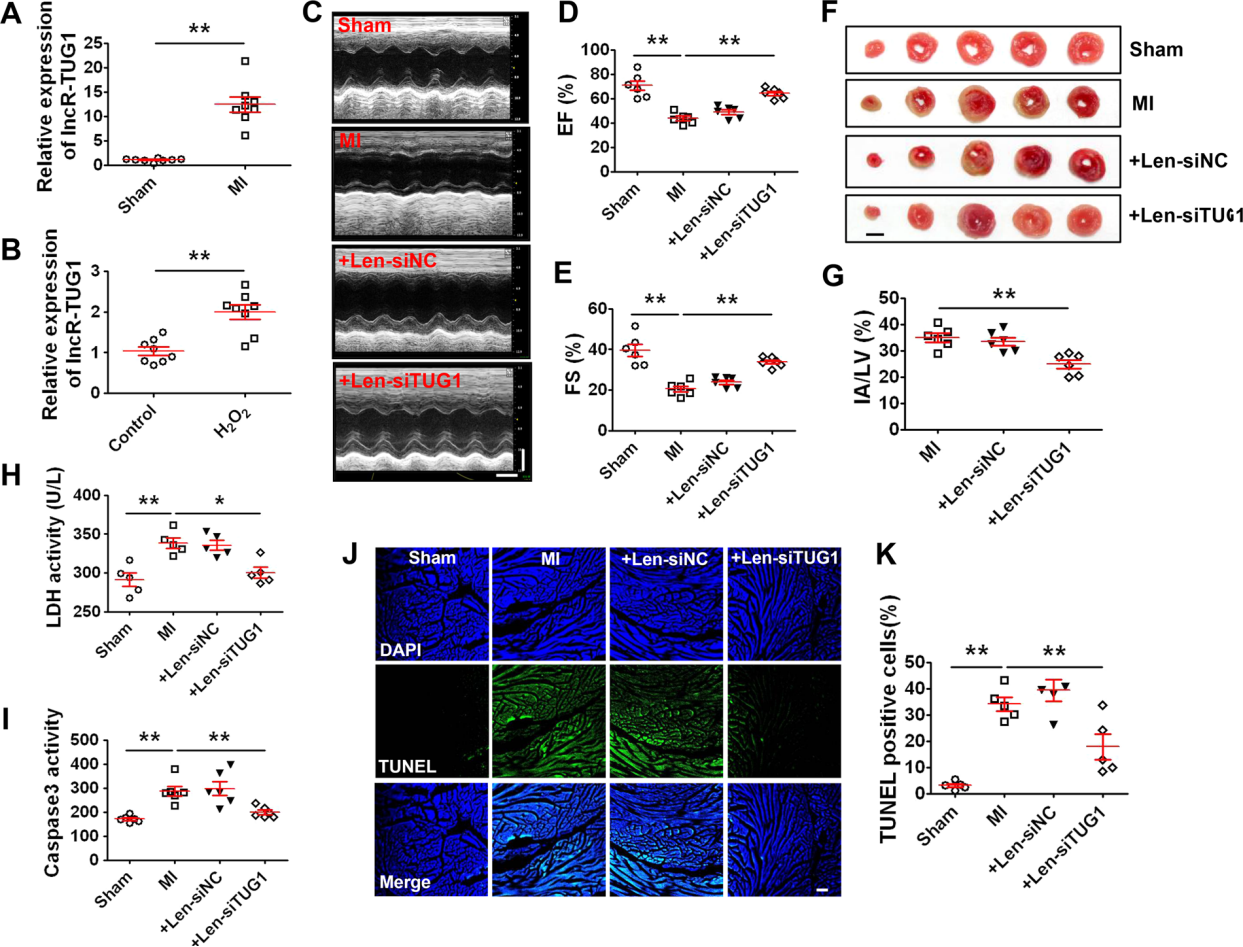
Silencing of lnR-TUG1 attenuates ischemic injury of MI mice. **a** Real-time RT-PCR data showing the upregulation of lncR-TUG1 in heart tissue of MI mice compared with sham mice (*n* = 8). **P < 0.01 by Student’s *t*-test. Data are presented as mean ± SEM. **b** Expression upregulation of lncR-TUG1 in NRVMs treated with 200 μM H_2_O_2_ for 4 h (*n* = 8). **P < 0.01 by Student’s *t*-test. Data are presented as mean ± SEM. **c** Representative M-mode echocardiographic tracings (time stamps, 100 ms). Calibration bar: 2 mm. Echocardiographic assessment of the ejection fractions (EF) percentage (**d**) and fractional shortening (FS) (**e**) in hearts of sham or MI (3 day) mice infected with the Len-siNC or Len-siTUG1 (*n* = 6). **P < 0.01 by one-way ANOVA analysis with Tukey’s multiple comparison test. Data are presented as mean ± SEM. **f** Representative images showing infarct areas in cross section slices in MI (3 day) mice (scale bar: 3 mm). **g** Statistical analysis of IA/LV ratio. IA infarct area, LV left ventricle (*n* = 6). **P < 0.01 by Student’s *t*-test. Data are presented as mean ± SEM. **h** Increase in serum lactate dehydrogenase (LDH) activity in MI (3 day) mice and restoration by Len-siTUG1 administration (*n* = 5). *P < 0.05, **P < 0.01 by one-way ANOVA analysis with Tukey’s multiple comparison test. Data are presented as mean ± SEM. **i** Effect of Len-siTUG1 on caspase-3 activities in MI (3 day) mice. **P < 0.01 versus Sham (*n* = 6). **P < 0.01 by one-way ANOVA analysis with Tukey’s multiple comparison test. Data are presented as mean ± SEM. **j** Representative images of TUNEL staining for DNA defragmentation showing the apoptotic cells in MI (3 day) mice (nucleus stained in blue with DAPI and apoptotic cells stained in green) (scale bar: 60 μm). **k** The percentage of TUNEL-positive cell (*n* = 5). **P < 0.01 by one-way ANOVA analysis with Tukey’s multiple comparison test. Data are presented as mean ± SEM.

As the first step towards understanding the potential pathophysiological role of lncR-TUG1 in MI, we detected lncR-TUG1 expression in a mouse MI model and in a cellular model of oxidative insult, a key deleterious factor of ischemic injuries. The expression of lncR-TUG1 in the peri-infarct area was significantly increased three days post-MI, compared with sham-operated animals (Fig. 1a). H_2_O_2_ has been well documented to induce cardiomyocyte apoptosis^36^. In this study, 200 μM H_2_O_2_ treated for 4 h was used to induce cardiomyocytes apoptosis in neonatal rat ventricular myocytes (NRVMs). Consistent with the vivo results, lncR-TUG1 level was also markedly elevated relative to non-treated control cells in NRVMs pretreated with 200 μM H_2_O_2_ for 4 h (Fig. 1b).

To elucidate the vivo effects of lncR-TUG1 on MI injury, the lentiviral vectors carrying siTUG1 (Len-siTUG1) were injected into left ventricular chamber of mice to silence endogenous lncR-TUG1, lncR-TUG1 levels were significantly decreased in mice heart after len-siTUG1 administration on days 1, 2, and 3 (Fig. S1A). Futher, we injected Len-siTUG1 into left ventricular chamber of mice and established MI model for 3 days. 3 days post-MI, echocardiography examination showed that EF and FS were both significantly decreased in MI hearts, indicating the impaired cardiac function (Fig. 1c–e). These deleterious alterations were significantly attenuated. Triphenyltetrazolium chloride (TTC) staining showed that Len-siTUG1 significantly reduced the infarct size, whereas the Len-siNC did not elicit any appreciable changes (Fig. 1f, g). Moreover, lncR-TUG1 silencing significantly inhibited MI-induced elevation of lactate dehydrogenase (LDH) activity in serum and caspase-3 activity myocardium of MI mice (Fig. 1h, i). Furthermore, it was observed that the percentage of TUNEL-positive cells was significantly increased in the border zone of MI mice hearts, which was diminished by Len-siTUG1 (Fig. 1j, k).

### LncRNA-TUG1 acts as a ceRNA of miR-9 to promote cardiomyocyte apoptosis

**Fig. 2:**
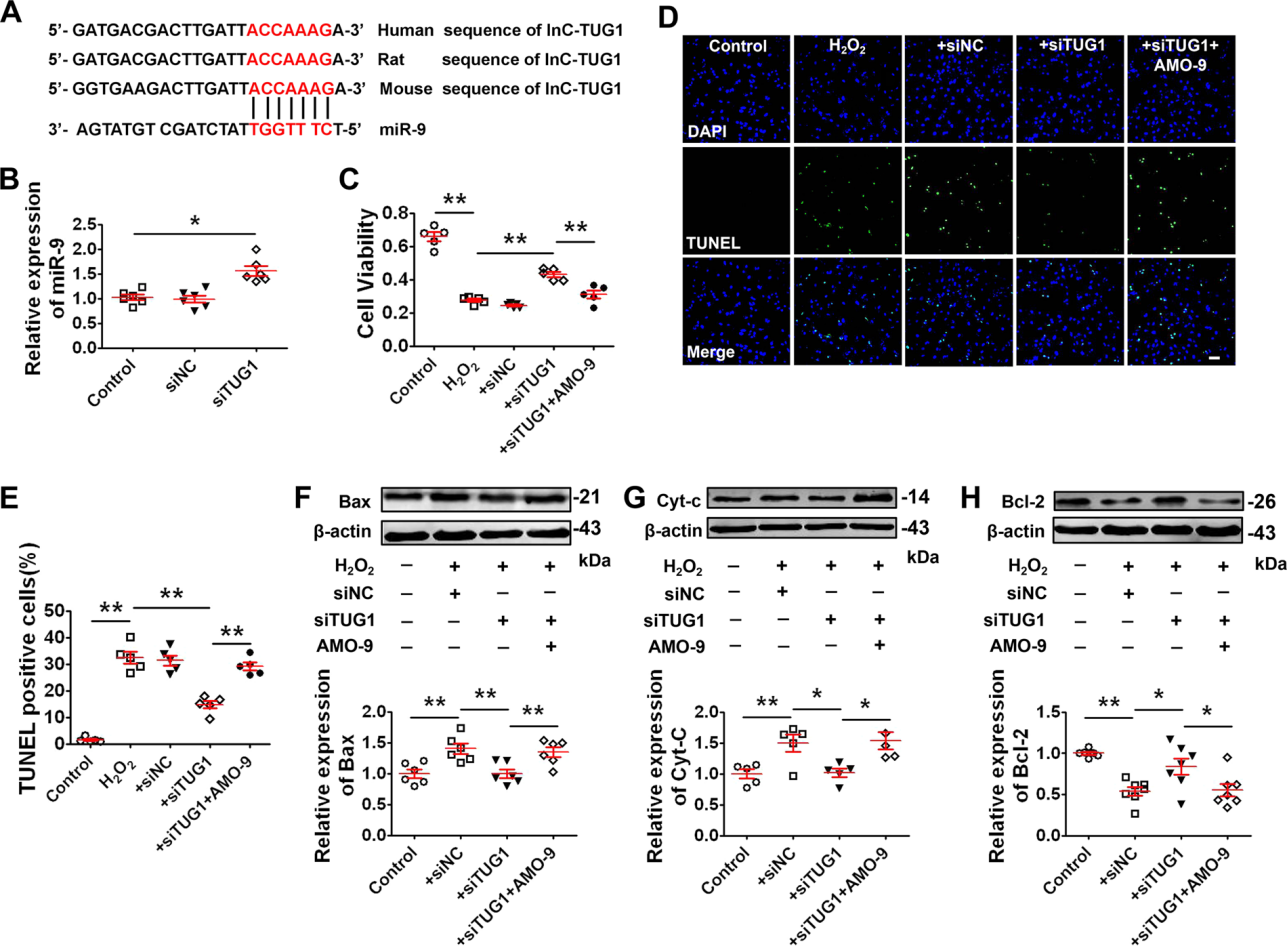
Silencing lncR-TUG1 alleviates H_2_O_2_-induced cardiomyocytes apoptosis by targeting miR-9. **a** The bioinformatics analysis showing the binding site for miR-9 with lncR-TUG1. **b** Increase in miR-9 level after LncR-TUG1 knockdown by siRNA (*n* = 6). *P < 0.05 by Student’s *t*-test. Data are presented as mean ± SEM. NRVMs were divided into five groups: control, H_2_O_2_ treatment, H_2_O_2_ + siNC, H_2_O_2_ + siTUG1, and H_2_O_2_ + siTUG1 + AMO-9. **c** Alterations of cell viability of NRVMs by MTT assay (*n* = 5). **P < 0.01 by one-way ANOVA analysis with Tukey’s multiple comparison test. Data are presented as mean ± SEM. **d** Representative images of TUNEL staining of NRVMs for DNA defragmentation showing the apoptotic cells (scale bar: 60 μm). **e** Statistical results of TUNEL-positive cells per field (*n* = 5). **P < 0.01 by one-way ANOVA analysis with Tukey’s multiple comparison test. Data are presented as mean ± SEM. **f**–**h** Western blot analysis of protein levels of Bax (*n* = 6), cytochrome-c (Cyt-c; *n* = 5), and Bcl-2 (*n* = 7) in NRVMs with different treatments. *P < 0.05, **P < 0.01 by one-way ANOVA analysis with Tukey’s multiple comparison test. Data are presented as mean ± SEM.

Recent studies have suggested that lncRNA can act as a competing endogenous RNA (ceRNA) or a molecular sponge to limit the availability of functional miRNAs by the sequence complementary mechanism^37^. Indeed, the direct interaction between lncR-TUG1 and miR-9 has been characterized in breast cancer^28^ and ischemic stroke^15^. To determine whether miR-9 is involved in the regulatory effect of lncR-TUG1 in MI, we used siRNA technology to silence lncR-TUG1 gene in vitro, and found that lncR-TUG1 expression was significantly reduced in NRVMs after siTUG1–2 transfection (Fig. S1B). So we chose siTUG1–2 for subsequent experiments. Bioinformatics analysis of miRNA recognition sequences was performed and the result revealed the present of a binding site of miR-9 in the lncR-TUG1 sequence with high interspecies conservation (Fig. 2a). Downregulation of lncR-TUG1 significantly increased miR-9 level (Fig. 2b). We then examined whether miR-9 mediated the pro-apoptotic effect of lncR-TUG1 in our experimental models. Results showed that downregulation of lncR-TUG1 reversed the effects of H_2_O_2_ and contributed to prevent cell apoptosis. However, these beneficial effects of lncR-TUG1 downregulation were reversed by AMO-9 (Fig. 2c–e). Furthermore, H_2_O_2_ remarkably increased the expression of pro-apoptotic proteins, including Bax (Fig. 2f) and cytochrome-c (Cyt-c; Fig. 2g), and suppressed the anti-apoptotic protein Bcl-2 expression (Fig. 2h). On the contrary, downregulation of lncR-TUG1 reversed above alterations. More importantly, the effects of lncR-TUG1 inhibition on apoptotic proteins were diminished by silencing miR-9 (Fig. 2f–h). In addition, we validate the effect of the another construct siTUG1-1 in cardiomyocyte apoptosis. Since the siTUG1-1 transfection efficiency has not been reduced less than 50% (Fig. S1B), we altered the siTUG1-1 transfection concentration. LncR-TUG1 expression was significantly reduced less than 50% in NRVMs after siTUG1-1 transfection with 100nmol/L (Fig. S2A) and downregulation of lncR-TUG1 significantly increased miR-9 level (Fig. S2B). We then examined whether siTUG1-1 can exert similar pro-apoptotic effect with siTUG1-2. Results showed that downregulation of lncR-TUG1 by siTUG1-1 reversed the effects of H_2_O_2_, contributed to prevent cell apoptosis and reversed Bax/Bcl-2 expression alterations induced by H_2_O_2_ (Fig. S2C–G). Meanwhile, the effects of lncR-TUG1 inhibition were diminished by silencing miR-9 (Fig. S2C–G). Taken together, these data suggest that lncR-TUG1 targets miR-9 and regulates ischemic cardiomyocyte apoptosis.

### The cardio-protective role of miR-9 in cardiac injury during MI

**Fig. 3:**
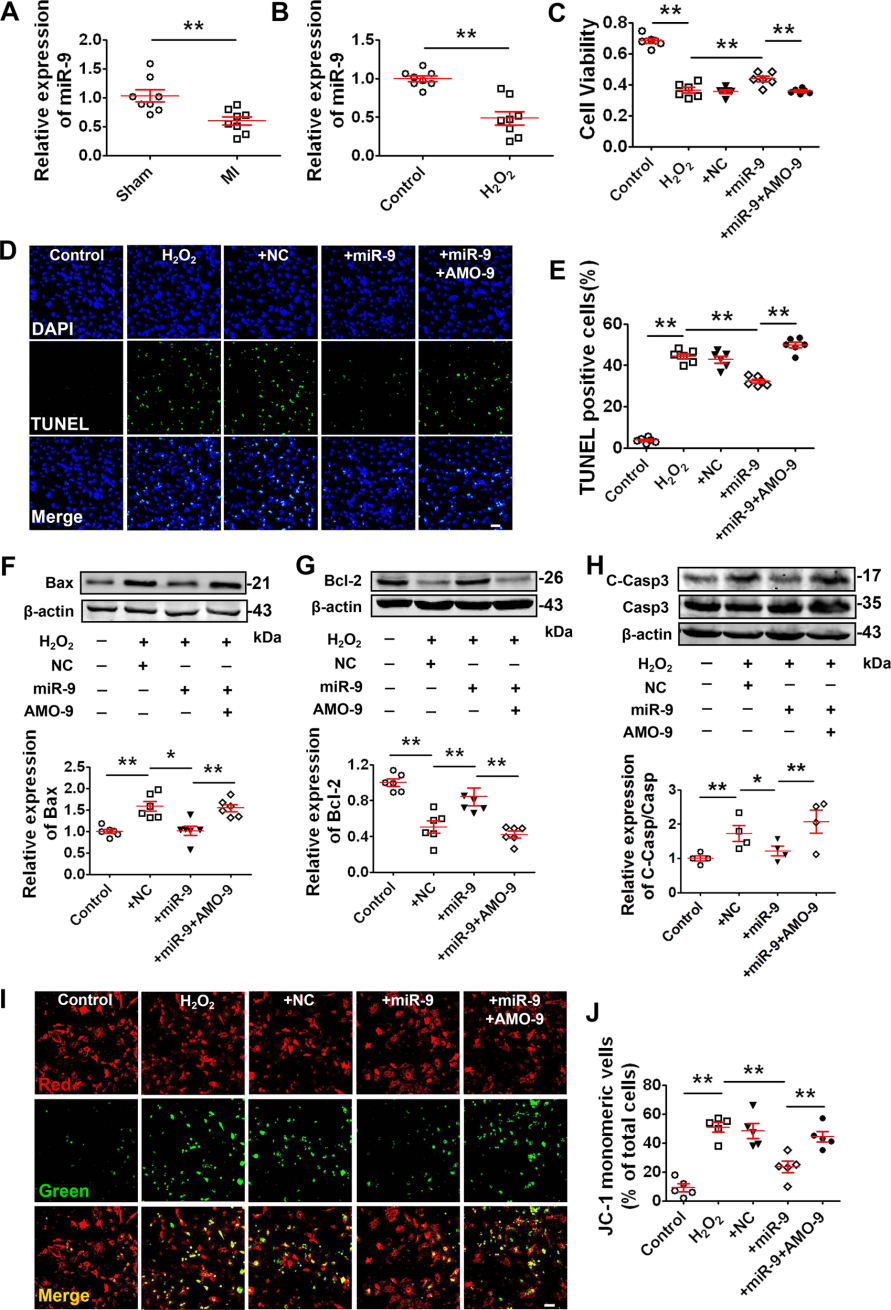
miR-9 prevents cardiomyocyte apoptosis in response to H_2_O_2_. **a** Downregulation of miR-9 level in MI mice heart (*n* = 8). **P < 0.01 by Student’s *t*-test. Data are presented as mean ± SEM. **b** Elevation of miR-9 level in NRVMs treated with 200 μM H_2_O_2_ for 4 h (*n* = 8). **P < 0.01 by Student’s *t*-test. Data are presented as mean ± SEM. NRVMs were transfected with NC (negative control), miR-9, AMO-9, or miR-9 + AMO-9 for 24 h, then treated with H_2_O_2_ (200 μM) for 4 h. **c** Restoration of cell viability by miR-9 in NRVMs treated with 200 μM H_2_O_2_ for 4 h, determined MTT assay (*n* = 6). **P < 0.01 by one-way ANOVA analysis with Tukey’s multiple comparison test. Data are presented as mean ± SEM. **d** Representative images of TUNEL staining of NRVMs for DNA defragmentation showing the apoptotic cells (scale bar: 60 μm). **e** Statistical results of TUNEL-positive cells per field indicating the suppression of H_2_O_2_-induced cell apoptosis by miR-9 (*n* = 6). **P < 0.01 by one-way ANOVA analysis with Tukey’s multiple comparison test. Data are presented as mean ± SEM. **f** Abolishment of H_2_O_2_-induced expression upregulation of Bax by miR-9 overexpression (*n* = 6). *P < 0.05, **P < 0.01 by one-way ANOVA analysis with Tukey’s multiple comparison test. Data are presented as mean ± SEM. **g** Abolishment of H_2_O_2_-induced expression downregulation of Bcl-2 by miR-9 overexpression (*n* = 6). **P < 0.01 by one-way ANOVA analysis with Tukey’s multiple comparison test. Data are presented as mean ± SEM. **h** Decrease of cleaved-caspase-3 (C-Casp3) by miR-9 in the presence of H_2_O_2_ (*n* = 4). *P < 0.05 by one-way ANOVA analysis with Tukey’s multiple comparison test. Data are presented as mean ± SEM. **i** Upper panels show representative fluorescent images of JC-1 monomeric mitochondria showing green fluorescence and JC-1 aggregated mitochondria from Control, H_2_O_2_, H_2_O_2_ + NC, H_2_O_2_ + miR-9, and H_2_O_2_ + miR-9 + AMO-9 groups (scale bar: 60 μm). **j** Reduction of H_2_O_2_-induced JC-1 staining by miR-9 overexpression (*n* = 5). **P < 0.01 by one-way ANOVA analysis with Tukey’s multiple comparison test. Data are presented as mean ± SEM.

Based on the above results, we subsequently evaluated the effects of miR-9 on cardiomyocyte apoptosis. Three days post-MI, the expression of miR-9 in the peri-infarct area of mice hearts was significantly decreased compared with sham-operated animals (Fig. 3a). Meanwhile, the expression of miR-9 in H_2_O_2_-treated NRVMs was lower than that in non-treated control cells (Fig. 3b**)**. MiR-9 or AMO-9 was transfected into NRVMs to overexpress or knockdown miR-9 expression (Fig. S3A, B). Overexpression of miR-9 inhibited the H_2_O_2_-induced decrease of cell viability and increase in cell apoptosis (Fig. 3c–e). However, we also found that these beneficial effects of miR-9 overexpression were reversed by AMO-9 (Fig. 3c–e). These results indicate that the downregulation of miR-9 expression contributed to cardiomyocyte apoptosis and miR-9 overexpression reversed apoptosis induced by H_2_O_2_. We further investigated the potential role of miR-9 in regulating the mitochondrial apoptotic pathway by first assessing the changes of expression of the apoptosis-related genes. MiR-9 remarkably prevented the upregulation of Bax and downregulation of Bcl-2 in NRVMs treated with H_2_O_2_ (Fig. 3f, g). The ratio of cleaved/activated form of caspase-3 over total caspase-3 proteins was markedly increased by oxidative stress, which was diminished by miR-9 overexpression (Fig. 3h). Notably, the anti-apoptotic effect of miR-9 was counteracted by AMO-9 (Fig. 3f–h).

It is known that in apoptotic cells, the loss of mitochondrial membrane potential **(**Δψm**)** causes the formation of JC-1 monomeric mitochondria that can be monitored by changes of fluorescence dyes. The ratio of green to red fluorescence was used to quantify the Δψm, with a low ratio representing mitochondrial depolarization. In our study, H_2_O_2_ treatment caused substantial formation of monomeric JC-1, indicative of a loss of Δψm (Fig. 3i, j). Transfection of miR-9 rescued the lost Δψm as indicated by the abrogation of the H_2_O_2_-induced formation of JC-1 monomers (Fig. 3i, j).

**Fig. 4:**
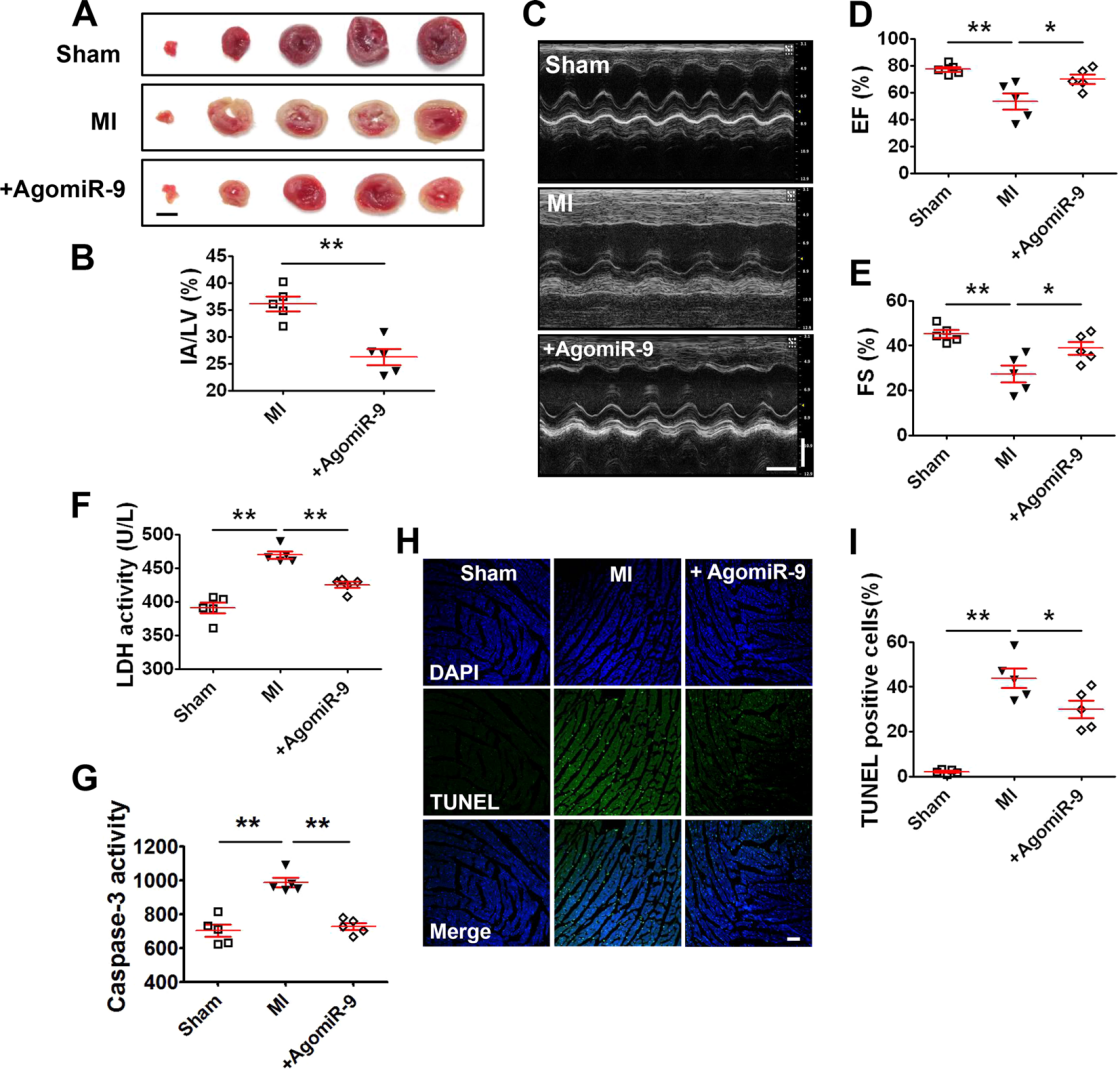
miR-9 attenuates ischemic injury of MI mice. **a** Representative images of cross section slices showing infarct areas in MI (3 day) mice (scale bar: 3 mm). **b** Statistical analysis of IA/LV ratio. IA: infarct area; LV: left ventricle (*n* = 5). **P < 0.01 by Student’s *t*-test. **c** Representative M-mode echocardiographic tracings in MI (3 day) mice (time scale for *X*-axis: 100 ms; scale bar for *Y*-axis: 2 mm). Data are presented as mean ± SEM. **d** Ejection fraction (EF) and **e** Fractional shortening (FS) (*n* = 5). *P < 0.05, **P < 0.01 by one-way ANOVA analysis with Tukey’s multiple comparison test. Data are presented as mean ± SEM. **f** Effects of agomiR-9 on serum lactate dehydrogenase (LDH) activity in MI (3 day) mice (*n* = 5). **P < 0.01 by one-way ANOVA analysis with Tukey’s multiple comparison test. Data are presented as mean ± SEM. **g** Inhibition of caspase-3 activity by agomiR-9 in MI (3 day) mice (*n* = 5). **P < 0.01 by one-way ANOVA analysis with Tukey’s multiple comparison test. Data are presented as mean ± SEM. **h** Effects of agomiR-9 on cardiac apoptosis evaluated by TUNEL staining in MI (3 day) mice (scale bar: 60 μm). **i** The percentage of TUNEL-positive cell in different groups (*n* = 5). *P < 0.05, **P < 0.01 by one-way ANOVA analysis with Tukey’s multiple comparison test. Data are presented as mean ± SEM.

To elucidate the in vivo effects of miR-9 overexpression on MI (3 day) injury, we directly injected agomiR-9 into left ventricular chamber of mice before the onset of MI procedure. MiR-9 levels were significantly increased in mice heart after agomiR-9 administration on days 1, 2, and 3 (Fig. S3C). Myocardial infarct size was significantly smaller in mice treated with agomiR-9 than in MI mice (Fig. 4a, b). Overexpression of miR-9 attenuated the deterioration of left ventricular performance as indicated by the increased EF% and FS% (Fig. 4c–e). In addition, miR-9 significantly inhibited ischemia-induced elevation of serum LDH level and myocardial caspase-3 activity in mice (Fig. 4f, g). Furthermore, the apoptotic ratio of MI mice significantly increased in comparison with sham controls. Overexpression of miR-9 reduced apoptosis of cardiac myocytes in the peri-infarct area of MI hearts (Fig. 4h, i).

### KLF5 as a target gene mediating the apoptosis-regulating property of miR-9

**Fig. 5:**
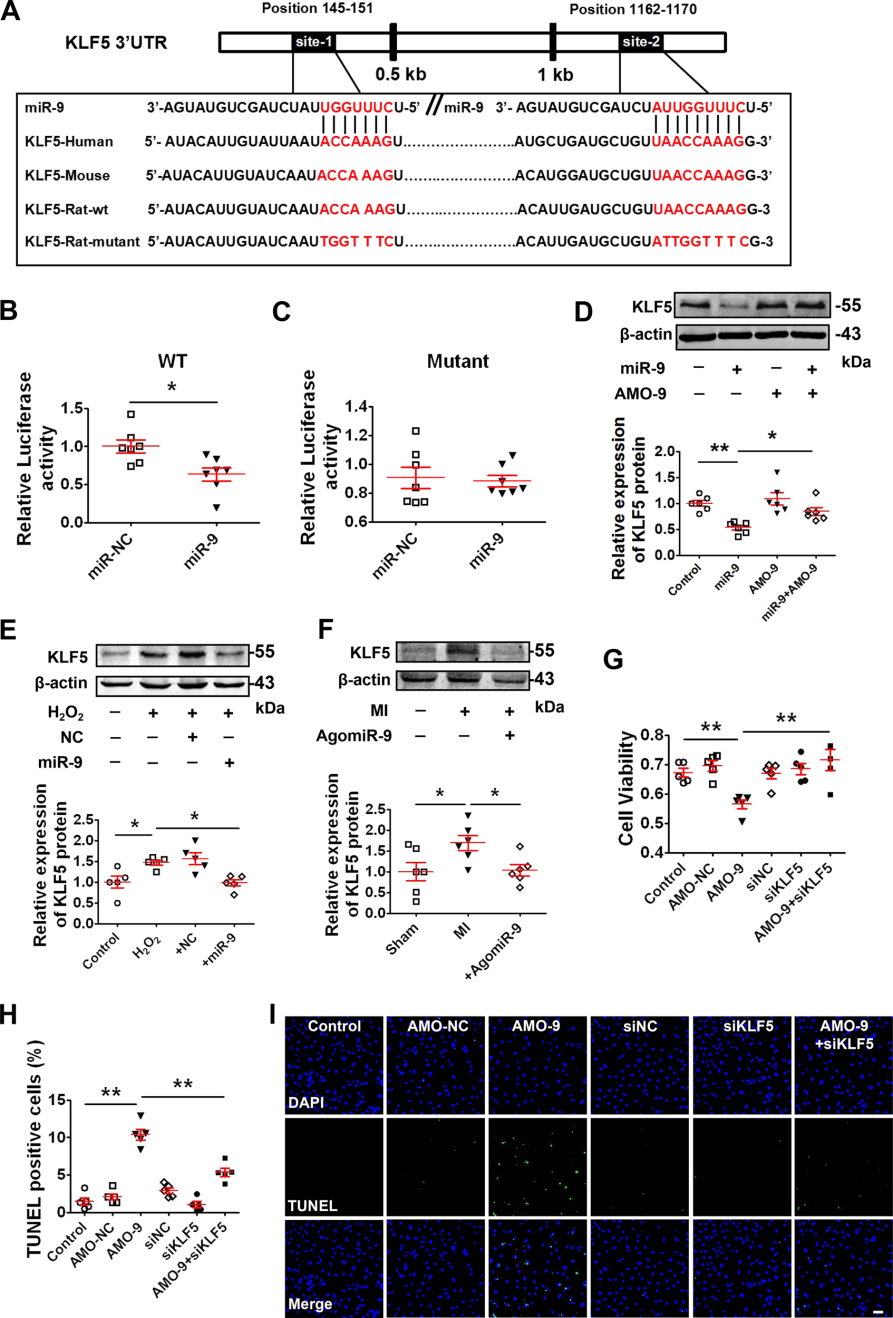
miR-9 regulates cardiomyocytes apoptosis by KLF5. **a** Alignment of miR-9 and the 3’UTR of KLF5 mRNA sequences showing the seed site complementarity and the nucleotide replacement mutations. **b**, **c** Dual Luciferase reporter assay for evaluating the effects of miR-9 mimic on luciferase activities elicited by the vector containing WT or mutant 3’UTR of rat KLF5 in HEK293T cells. MiR-9 overexpression markedly decreased the relative luciferase activity in the WT 3’UTR but not mutant 3’UTR of KLF5 mRNA (*n* = 7). *P < 0.05 by Student’s *t*-test. Data are presented as mean ± SEM. **d** Western blot analysis (*n* = 6) for KLF5 protein level, respectively, in cardiomyocytes transfected with miR-9, AMO-9 or co-transfection of miR-9 and AMO-9. *P < 0.05, **P < 0.01 by one-way ANOVA analysis with Tukey’s multiple comparison test. Data are presented as mean ± SEM. **e** Abrogation of H_2_O_2_-induced upregulation of KLF5 protein level by miR-9 overexpression in NRVMs (*n* = 5). *P < 0.05 by one-way ANOVA analysis with Tukey’s multiple comparison test. Data are presented as mean ± SEM. **f** Diminishment of abnormal upregulation of KLF5 protein level (*n* = 6) by miR-9 overexpression in MI heart. *P < 0.05 by one-way ANOVA analysis with Tukey’s multiple comparison test. Data are presented as mean ± SEM. **g** Restoration of reduced cell viability by silencing KLF5 in the presence of AMO-9 in NRVMs, as determined by MTT assay (*n* = 5). **P < 0.01 by one-way ANOVA analysis with Tukey’s multiple comparison test. Data are presented as mean ± SEM. **h** Statistical results of TUNEL-positive cells per field (*n* = 5 batches of cells). **P < 0.01 by one-way ANOVA analysis with Tukey’s multiple comparison test. Data are presented as mean ± SEM. **I** Representative images of TUNEL staining of NRVMs showing the apoptotic cells (scale bar: 60 μm).

To elucidate the target mechanisms by which miR-9 elicits its anti-apoptosis action in response to ischemia and oxidative stress, we first searched miRNAs databases for the potential target genes of miR-9 using the Targetscan computational miRNA target prediction algorithms (http://targetscan.org, Release 5.1). We identified Krüppel-like factor 5 (KLF5) as a candidate as its 3’UTR contains two domains matching the seed sequence of miR-9 (UTR NM_053394.2; Fig. 5a). These complementary sequences are evolutionarily conserved among the human, mouse and rat mRNAs. Luciferase reporter vectors containing the 3’UTR fragment of KLF5 encompassing the miR-9 binding sites or a mutated fragment were constructed and transfected into HEK293 cells. We found that miR-9 significantly inhibited the luciferase activity of the vector containing the wild-type binding site, whereas it failed to affect the luciferase activity elicited by the mutated construct (Fig. 5b, c), suggesting that miR-9 represses KLF5 by physically binding to the 3’UTR of this gene.

To confirm the regulatory effect of miR-9 on KLF5 in cardiomyocytes, we examined the changes in KLF5 protein level following miR-9 treatment. Western blot (Fig. 5d) analyses indicated that miR-9 significantly repressed the expression of KLF5 protein level, which was reversed by co-transfection with AMO-9. In contrast, AMO-9 markedly increased protein expression of KLF5 (Fig. 5d). On the other hand, H_2_O_2_ treatment significantly increased KLF5 level, which was inversely correlated with miR-9 level in cardiomyocytes (Fig. 5e). Moreover, the protein level of KLF5 was significantly higher three days post-MI in hearts from the MI group than in those from the sham group (Fig. 5f). AgomiR-9 abolished the expression upregulation of KLF5 induced by MI injury (Fig. 5f).

If repression of miR-9 indeed mediates the process of cardiomyocyte apoptosis induced by MI/oxidative stress, then inhibition of miR-9 is anticipated to produce similar effects as H_2_O_2_. Similarly, silence of KLF5 without altering miR-9 level is expected to produce similar effects as miR-9. To examine this notion, we investigated whether silence of KLF5 by siRNA (siKLF5) could prevent cardiomyocyte apoptosis in response to AMO-9 or H_2_O_2_. As illustrated in Fig. S4A, B, the mRNA and protein level of KLF5 were reduced by nearly 50% in NRVMs transfected with siKLF5-1. We chose siKLF5-1 for the subsequent experiments. AMO-9 decreased cell viability and induced cell apoptosis (Fig. 5g–i). On the other hand, siKLF5 rescued the decreased cell viability (Fig. 5g) and mitigated the increased apoptosis induced by AMO-9 (Fig. 5h, i). In addition, we validate the effect of the another construct siKLF5-2. KLF5 expression was significantly reduced less than 50% in NRVMs after siKLF5-2 transfection with 100nmol/L (Fig. S4C) and suppressed the decrease of cell viability induced by H_2_O_2_ (Fig. S4D).

**Fig. 6:**
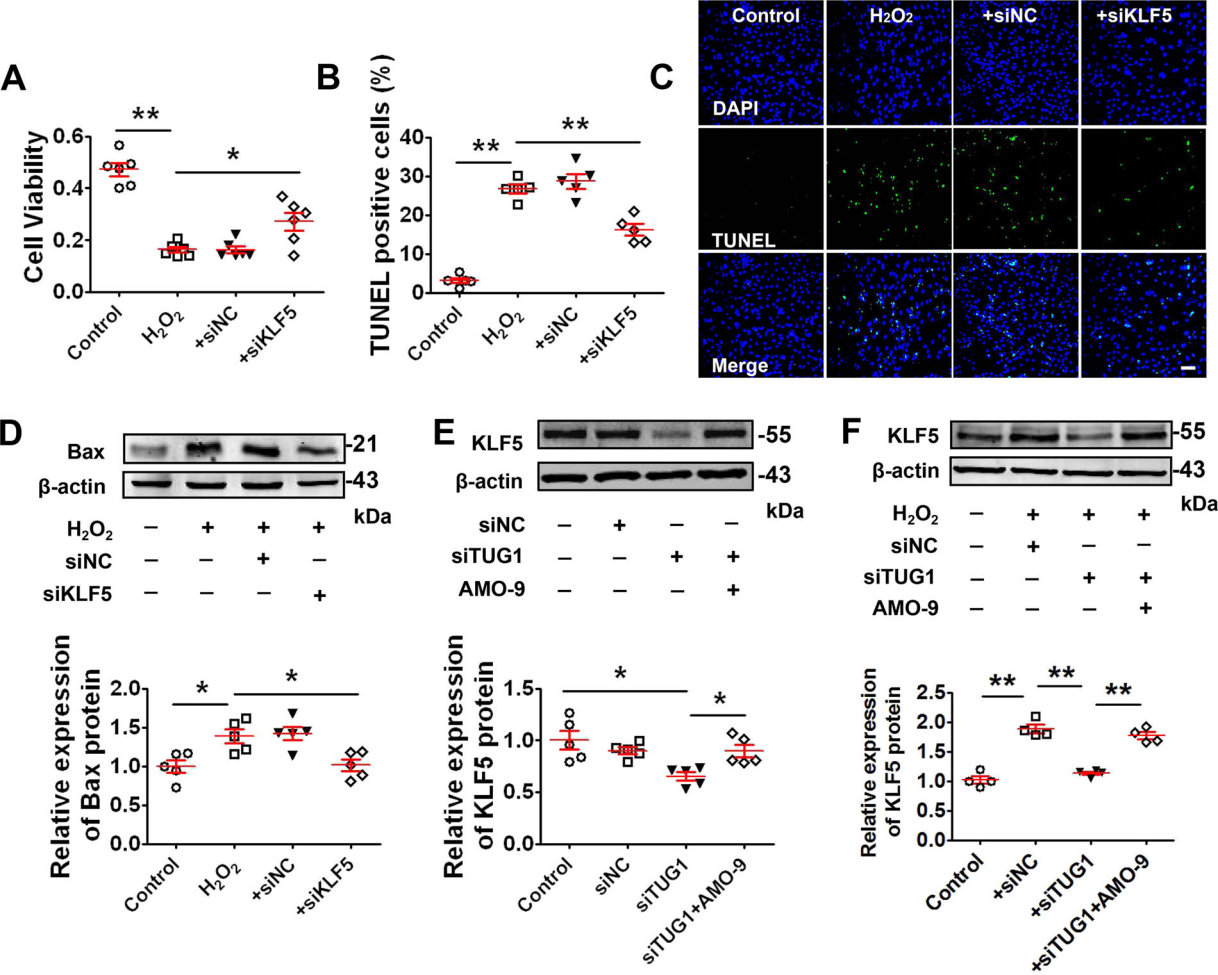
KLF5 silencing inhibits cardiomyocyte apoptosis. **a** Restoration of reduced cell viability by silencing KLF5 in NRVMs treated with 200 μM H_2_O_2_ for 4 h (*n* = 6). *P < 0.05, **P < 0.01 by one-way ANOVA analysis with Tukey’s multiple comparison test. Data are presented as mean ± SEM. **b** Statistical results of TUNEL-positive cells per field (*n* = 5). **P < 0.01 by one-way ANOVA analysis with Tukey’s multiple comparison test. Data are presented as mean ± SEM. **c** Representative images of TUNEL staining of NRVMs for DNA defragmentation showing the apoptotic cells (scale bar: 60 μm). Data are presented as mean ± SEM. **d** KLF5 silencing suppressed Bax elevation in NRVMs treated with H_2_O_2_ (*n* = 5). *P < 0.05 by one-way ANOVA analysis with Tukey’s multiple comparison test. Data are presented as mean ± SEM. **e** Downregulation of KLF5 protein level by LncR-TUG1 knockdown in NRVMs (*n* = 5). Note that the effect of LncR-TUG1 knockdown was prevented by AMO-9. *P < 0.05 by one-way ANOVA analysis with Tukey’s multiple comparison test. Data are presented as mean ± SEM. **f** Decrease of H_2_O_2_-induced upregulation of KLF5 protein level by LncR-TUG1 knockdown in NRVMs (*n* = 4). **P < 0.01 by one-way ANOVA analysis with Tukey’s multiple comparison test. Data are presented as mean ± SEM.

Moreover, the data in Fig. 6a–c revealed that siKLF5 was effective in suppressing the H_2_O_2_-induced decrease of cell viability and apoptotic cell death. In addition, the Bax expression was enhanced by H_2_O_2_, which was inhibited by KLF5 silencing (Fig. 6d). Furthermore, knockdown of lncR-TUG1 by siRNA markedly reduced KLF5 expression and knockdown of miR-9 by AMO-9 counteracted the effect of lncR-TUG1 on KLF5 expression (Fig. 6e). In addition, lncR-TUG1 silencing remarkably prevented H_2_O_2_ treatment induced upregulation of KLF5, which was also diminished by AMO-9 (Fig. 6f). These results indicated that KLF5 mediates the actions of lncR-TUG1 and miR-9 during cardiac apoptosis.

## Discussion

Myocardial infarction (MI) is a common response to a variety of physiological as well as pathological stimuli and it can eventually lead to heart failure. Inhibition of apoptosis is considered to be a therapeutic strategy for the management of MI. It is essential to discover appropriate therapeutic targets to suppress cardiomyocyte apoptosis and the associated cardiac injury. The aims of the present study were to characterize the pathophysiological role of the lncR-TUG1/miR-9 axis in cardiac injuries produced by ischemic and oxidative insults and to delineate the cellular/molecular mechanisms for the function of this axis. Our experimentations generated several new findings. First, there is inverse relationship between lncR-TUG1 and miR-9 in terms of their expression, with the former upregulated and the latter downregulated, in infarct myocardium of mice and in oxidative cardiomyocytes isolated from rats. Second, the deregulation of lncR-TUG1/miR-9 contributes significantly to the impairment of cardiac function and cardiac injuries (infarct size) under ischemia. Third, promotion of cardiomyocyte apoptosis is likely a cellular mechanism underlying the detrimental effects of the lncR-TUG1/miR-9 axis and stimulation of the mitochondrial death pathway is an important signaling mechanism for lncR-TUG1/miR-9 to promote the apoptotic cell death induced by MI and oxidative stress. Forth, KLF5, as a target gene of miR-9, likely mediates the pro-apoptotic action of the lncR-TUG1/miR-9 axis. And fifth, lncRNA acts as a ceRNA by sequence complementarity to limit the functional availability of miR-9; in other words, lncR-TUG1 inhibits miR-9 to elicit pro-apoptotic action and miR-9 by its own produces anti-apoptotic effect. Our study has therefore unraveled novel aspects of the cellular functions and pathophysiological roles of lncR-TUG1 and miR-9, both of which could be considered potential molecular targets for treatment of cardiac damages associated with apoptosis as that seen with ischemic and oxidative stress. It is worth noting that lncR-TUG1 is a highly conserved in mammals including rat and mouse, which were both involved in our study, e.g., the sequence of lncR-TUG1 in rat is almost totally same with in mouse, and above 70% overlapped with in human.

Recently, several studies have revealed that lncRNAs play a critical role in the regulation of cardiac ischemia. For example, Liu et al. demonstrated that lncRNA-CAIF upregulation represses autophagic cell death and attenuates MI by inhibiting p53-mediated myocardin expression^38^. Zhang et al. reported that MI-induced lncRNA ZFAS1 activation leads to intracellular Ca^2+^ overload in cardiomyocytes and weakened the contractility of cardiac muscles by directly binding SERCA2a protein^39^. Wang et al. found that lncRNA-NRF regulates programmed necrosis and myocardial injury during ischemia and reperfusion by targeting miR-873^40^. Increasing studies indicate the importance of a new regulatory mechanism by which lncRNAs function as a ceRNA to inhibit endogenous miRNAs via interfering with miRNAs, thus reducing the expressions of miRNA targets^41^. It is reported that lncR-TUG1 can influence tumorigenesis in several cancers through targeting miR-9^13,42^. Importantly, in this study, we found that both lncR-TUG1 and KLF5 contain the binding site of miR-9 and revealed that miR-9 can directly bind to KLF5 in a sequence-specific manner. We therefore speculate that the upregulation of lncR-TUG1 during MI competitively binds to miR-9 and releases the inhibitory effects of miR-9 on KLF5, which results in the upregulation of KLF5 and promotes cardiomyocytes apoptosis.

MiR-9 is a brain-enriched miRNA, which participated in regulating neuronal development and tumor formation. Here, we have demonstrated for the first time that miR-9 protects against H_2_O_2_-induced mitochondrial dysfunction in NRVMs and prevents cardiac dysfunction and inhibits cardiomyocyte apoptosis in MI mice heart. However, it should been noticed that the effects of miR-9 on apoptosis are controversial. Zhu et al.^24^ found that overexpression of miR-9 significantly attenuates colorectal cancer cell proliferation and promotes cell apoptosis. It appears that the role of miR-9 in regulating cell apoptosis may be tissue/cell type specific.

KLF5 (also known as BTEB2 and IKLF), a member of the Kruppel-like family of transcription factors, binds to GC boxes at the promoter regions of a number of genes, regulating transcription and the signaling function during cell proliferation, apoptosis, migration, and differentiation^43,44^. KLF5 promotes apoptosis induced by TNF-α in prostate cancer cells via upregulating mitogen-activated protein kinase kinase 7 (MKK7)^45^. Moreover, KLF5 induces apoptosis via inducing pro-apoptotic protein Bax in esophageal cancer cells^34,46^. These results suggest that KLF5 may be a crucial gene involved in apoptosis. In this study, when KLF5 was silenced by siRNA, AMO-9 lost its ability to induce apoptosis (Fig. 5), indicating that KLF5 mediated the suppressing effect of miR-9 on apoptosis in cardiomyocytes. Our data further identified Bax as a downstream effector molecule inducing apoptosis (Fig. 6). Silence of lncR-TUG1 released miR-9 to repress KLF5 expression. These results suggest that KLF5 is a crucial gene mediating the apoptotic function of lncR-TUG1/miR-9. However, the exact mechanism by which KLF5 stimulates the mitochondrial death pathway needs further study.

## Materials and methods

### Animals

Healthy adult male Kunming mice (25–30 g) used in the present study were fed with food and water under standard animal room conditions (temperature 23 ± 1 °C; humidity 55 ± 5%). All animal care and experimental procedures were approved by the Animal Care and Use Committee of Harbin Medical University. All studies involving animals are reported in accordance with the Guide for the Care and Use of Laboratory Animals, published by the US National Institutes of Health (NIH Publication, 8th Edition, 2011). The investigator grouped the samples randomly. The another investigator carried out subsequent experiments and the investigator was blinded to the group allocation during the experiment. No statistical methods were used to estimate sample size.

### Cell culture

Cardiomyocytes were isolated from 1 to 3-day-old neonatal SD rats obtained from the Experimental Animal Center of the Second Affiliated Hospital of Harbin Medical University, China. Briefly, neonatal SD rats were anaesthetized by inhalation of 2% isoflurane and killed by cervical dislocation. Hearts were rapidly excised and cleaned with 75% ethanol. Then hearts were minced in serum-free DMEM, and then digested in 0.25% trypsin solution. Dispersed cells were suspended in DMEM containing 10% fetal bovine serum and 5% penicillin/streptomycin, followed by centrifugation. Finally, the cells were plated into culture flasks (non-coated). Cardiomyocytes were cultured with 5% CO_2_ at 37 °C for 2 h, and then plated onto 96-well plates or 6-well plates. Cardiomyocytes were tested for mycoplasma contamination once a month.

### Transfection of miRNA and siRNA

Cardiomyocytes (1 × 10^5^ per well) were starved in serum-free medium for 24 h before transfection. MiR-9 mimic (miR-9), miR-9 antisense inhibitor (AMO-9) and negative control (NC) were transfected by Opti-MEM^R^I (Invitrogen, Carlsbad, CA) and X-treme GENE siRNA transfection reagent (Roche, Penzberg, Germany) according to the manufacturer’s instructions. The concentration of siRNA and miR-9 tested was 50 nmol/L, and AMO-9 was 100 nmol/L. MiR-9 sequence is 5′-UCUUUGGUUAUCUAGCUGUAUGA-3′. MiR-9 inhibitor (AMO-9) sequence is 5′-UCAUACAGCUAGAUAACCAAAGA-3′. The NC sequence is 5′-UUUGUACUACACAAAAGUACUG-3′. The siTUG1-1 sequence is: 5′-GCAGTAATTFFAFTFFATA-3′. The siTUG1-2 sequence is: 5′-GGAUAUAACCAGAGAACAA-3′. The siTUG1-3 sequence is: 5′-CCTGATACCATCGATATAT-3′. The siKLF5-1 sequence is: 5′-CCTCCAGTTCCGATAATTT-3′. The siKLF5-2 sequence is: 5′-GCGCCAGAGGTGAACAATA-3′. The siKLF5-3 sequence is: 5′-CCGTCCTATGCTGCTACAA -3′.

### Cell viability assay

Cells (2 × 10^4^ per well) were seeded in a 96-well culture plate. Cell viability was measured by 3-(4,5-dimethylthiazol-2-yl)-2,5-diphenyltetrazolium bromide (MTT) assay according to the manufacturer’s instructions. The absorbance was measured at 490 nm by Microplate Reader (Infinite M200, TECAN; Salzburg, Austria).

### Mouse model of MI and RNA construct administration

Healthy adult male Kunming mice (25–30 g) were anesthetized with 2, 2,2-tribromoethanol (20 mg/kg) (Sigma, St. Louis, MO, USA) via i.p., and were then orally intubated with 20-gauge tube and ventilated (mouse ventilator, Kent Scientific corporation, Northwest Connecticut, USA) at a respiratory rate of 120 breaths/min and a tidal volume of 1.5 ml. A left thoracotomy was performed between the 3th and the 4th ribs to expose the heart. To evaluate the role of lncR-TUG1 in MI, lentiviral vectors carrying lncR-TUG1 siRNA (Len-siTUG1) (Genechem, Shanghai, China) or the lentivirus carrying its negative control construct (Len-siNC) was injected into the left ventricular cavity at a dosage of 1 × 10^7^ TU per mice followed by creation of a mouse MI model. In addition, agomiR-9 (Ribo-bio, Guangzhou, China), is the double-stranded RNA analogues identical to the mature miR-9a-5p (5′-UCUUUGGUUAUCUAGCUGUAUGA-3′), was chemically modified with methylation and cholesterol modification to ensure its long-lasting stability and enhanced cell membrane affinity in vivo. AgomiR-9 was injected into the left ventricular cavity through the tip of the heart with a 30-gauge syringe at a dosage of 200 nmol/kg at a volume of 80 μL 0.9% saline, and an equal volume of saline was administered to sham control mice. Then, aorta was occluded by a bulldog clamp for 10 seconds after injection. A segment of saline-soaked 8–0 sutures was then looped around the left anterior descending (LAD) coronary artery near its origin from the left coronary artery. The chest cavity was sutured by a segment of saline-soaked 5–0 sutures and the thorax was then closed. Sham-operated mice underwent an identical procedure except that the thread was passed through the myocardium without tying.

### Echocardiographic measurements

Three days post-MI, cardiac function was evaluated by transthoracic echocardiography with an ultrasound system (Panoview β1500, Cold Spring Biotech; Taiwan, China) equipped with a 30-MHz phased-array transducer. Left ventricular ejection fraction (EF%) and fractional shortening (FS%) were calculated from M-mode recording as described previously^47^. All animals were euthanized following echocardiographic measurements under anesthesia via cervical dislocation.

### TTC staining

Three days post-MI, the whole heart was removed and cut into 2-mm thick slices. The specimens were fixed with 4% paraformaldehyde for 24 h and then stained with 1% triphenyltetrazolium chloride (TTC) at 37 °C for 20 min. Infarct area is stainless while the non-infarct area is stained red. The left ventricle was separated and the ischemic area of the ventricle was dissected out. The infarct area was measured by the weight ratio of infarct area and left ventricle.

### LDH and Caspase-3 activity assays

LDH (Jiancheng Biotechnology Co., Nanjing, China) and caspase-3 (Beyotime, Shanghai, China) activities were measured using respective colorimetric assay kits according to the manufacturer’s’ instructions.

### Terminal deoxynucleotidyl transferase dUTP nick end labeling (TUNEL)

Apoptosis of mice heart was detected by staining ventricular specimens (ischemic border zone) three days post-MI and that of neonatal rat cardiomyocytes was determined with In situ Cell Death Detection Kit (TUNEL fluorescence FITC kit, Roche, Penzberg, Germany) according to the manufacturer’s instruction. After TUNEL staining, the ventricular specimens or cardiomyocytes were immerged into the DAPI (Sigma, St. Louis, MO, USA) solution to stain nuclei and apoptotic cells. Fluorescence staining was viewed under a laser scanning confocal microscope (Olympus, Fluoview1000; Tokyo, Japan).

### Real-time quantitative RT-PCR (qRT-PCR)

Total RNA from peri-infarct region of left ventricular myocardium or cultured neonatal cardiomyocytes of varying groups was extracted by using TRIZOL reagent (Invitrogen, Carlsbad, CA) according to the manufacturer’s protocols. After DNase I (Takara, Japan) treatment, RNA was reverse transcribed with reverse transcriptase (ReverTra Ace, Toyobo). The expression levels of miR-9, lncRTUG1 and KLF5 mRNA were determined using SYBR Green LightCycler480 Real-time PCR system (Roche, Penzberg, Germany), with U6 and GAPDH as internal controls for in-tube and inter-tube variabilities, as to be specified in appropriate sections. The sequences of primer pairs used are: U6 forward: 5′-GCTTCGGCAGCACATATACTAA-3′ and reverse: 5′-AACGCTTCACGAATTTGCGT-3′; rno-miR-9 forward: 5′-GCGGCGGTCTTTGGTTATCTAG-3′ and reverse: 5′-ATCCAGTGCAGGGTCCGAGG-3′; GAPDH forward: 5′-AAGAAGGTGGTGAAGCAGGC-3′ and reverse: 5′-TCCACCACCCAGTTGCTGTA-3′; KLF5 (rat) forward: 5′-AGCTCACCTGAGGACTCATA-3′ and reverse: 5′-GTGCGCAGTGCTCAGTTCT-3′; KLF5 (mouse) forward: 5′-ACCAGACGGCAGTAATGGACAC-3′ and reverse: 5′-ATTGTAGCGGCATAGGACGGAG-3′; lncR-TUG1 (rat) forward: 5′-TGGTGATTAGTTCAGGAATGGATG-3′ and reverse: 5′-TCAGGCACAGTTGGATACAGG-3′; and lncR-TUG1 (mouse) forward: 5′-TAACCATCTCACAAGGCTTCAAC-3′ and reverse: 5′-ACTCCCACTTCACTACTTCATCC -3′.

### Western blot analysis

Total protein samples were extracted from cultured neonatal cardiomyocytes after treatments and from peri-infarct region of left ventricular myocardium for immunoblotting analysis. Protein samples (100 µg) were fractionated by SDS-PAGE (12.5% polyacrylamide gels). The primary antibodies include Bcl-2 (1:1000; Cell Signaling; Danvers, MA, USA), Bax (1:1000; Proteintech, Chicago, USA), caspase-3 (1:500; Proteintech, Chicago, USA), and KLF5 (1:500; Proteintech, Chicago, USA). β-actin (1:1000; ZSJZ-Bio, Beijing, China) was used as an internal control. Images were captured on the Odyssey CLx Infrared Imaging System (LI-COR Biosciences, Lincoln, NE, USA). Western blot bands were quantified using Odyssey CLx v2.1 software (LI-COR Biosciences, Lincoln, NE, USA).

### Luciferase activity assay

Luciferase activity assay was performed using the Dual-Luciferase Reporter Assay System (Promega, Madison, WI, USA) according to the manufacturer’s instructions. HEK293 cells were seeded onto a 96-well culture plate and co-transfected with the 0.1 ng plasmid constructs (pGL3-KLF5–3′-UTR-WT or pGL3-KLF5–3′-UTR-mut, Ribo-bio, Guangzhou, China) and miR-9 mimic or mimic-NC at a concentration of 50 nmol/L using Opti-MEM^R^I (Invitrogen, Carlsbad, CA). Renilla luciferase was used as an internal control. Forty-eight hours after transfection, the cells were collected, and firefly and Renilla luciferase activities were evaluated.

### Data analysis

Data are presented as mean ± SEM. Student’s *t*-test was used for two group comparisons. One-way ANOVA (followed by Tukey-Kramer test) was used for multiple group comparisons. The data met the assumptions of the tests. The variance was similar between the groups that are being statistically compared. A value *P* < 0.05 was considered as a statistically significant difference. All samples data were used for statistics, no samples were excluded from the analysis.

## Supplementary information

Supplementary Material

Supplementary figure legends

## References

[1] Kajstura J (1996). Apoptotic and necrotic myocyte cell deaths are independent contributing variables of infarct size in rats. Lab. Invest..

[2] Saraste A (1997). Apoptosis in human acute myocardial infarction. Circulation.

[3] Gong C, Maquat LE (2011). lncRNAs transactivate STAU1-mediated mRNA decay by duplexing with 3’ UTRs via Alu elements. Nature.

[4] Clemson CM (2009). An architectural role for a nuclear noncoding RNA: NEAT1 RNA is essential for the structure of paraspeckles. Mol. Cell.

[5] Nahkuri S, Paro R (2012). The role of noncoding RNAs in chromatin regulation during differentiation. Wiley Interdiscip. Rev. Dev. Biol..

[6] Khaitan D (2011). The melanoma-upregulated long noncoding RNA SPRY4-IT1 modulates apoptosis and invasion. Cancer Res..

[7] Grote P (2013). The tissue-specific lncRNA Fendrr is an essential regulator of heart and body wall development in the mouse. Dev. Cell.

[8] Li X (2018). Loss of AZIN2 splice variant facilitates endogenous cardiac regeneration. Cardiovasc. Res..

[9] Young TL, Matsuda T, Cepko CL (2005). The noncoding RNA taurine upregulated gene 1 is required for differentiation of the murine retina. Curr. Biol..

[10] Zhang Q (2013). Down-regulation of long non-coding RNA TUG1 inhibits osteosarcoma cell proliferation and promotes apoptosis. Asian Pac. J. Cancer Prev..

[11] Xu Y (2015). Upregulation of the long noncoding RNA TUG1 promotes proliferation and migration of esophageal squamous cell carcinoma. Tumour Biol..

[12] Johnson R (2012). Long non-coding RNAs in Huntington’s disease neurodegeneration. Neurobiol. Dis..

[13] Li J, Zhang M, An G, Ma Q (2016). LncRNA TUG1 acts as a tumor suppressor in human glioma by promoting cell apoptosis. Exp. Biol. Med.(Maywood).

[14] Chen CCG, Yang X, Li C, Shi R, Zhao N (2016). Tanshinol suppresses endothelial cells apoptosis in mice with atherosclerosis via lncRNA TUG1 up-regulating the expression of miR-26a. Am. J. Transl. Res..

[15] Chen S (2017). LncRNA TUG1 sponges microRNA-9 to promote neurons apoptosis by up-regulated Bcl2l11 under ischemia. Biochem. Biophys. Res. Commun..

[16] Makeyev EV, Maniatis T (2008). Multilevel regulation of gene expression by microRNAs. Science.

[17] Bartel DP (2009). MicroRNAs: target recognition and regulatory functions. Cell.

[18] Filipowicz W, Bhattacharyya SN, Sonenberg N (2008). Mechanisms of post-transcriptional regulation by microRNAs: are the answers in sight?. Nat. Rev. Genet..

[19] Sun T (2017). The role of MicroRNAs in myocardial infarction: from molecular mechanism to clinical application. Int. J. Mol. Sci..

[20] Gao J, Ma X, Zhang Y, Guo M, Shi D (2017). The role of microRNAs in prethrombotic status associated with coronary artery disease. Thromb. Haemost..

[21] Zhao XB, Ren GS (2016). LncRNA taurine-upregulated gene 1 promotes cell proliferation by inhibiting MicroRNA-9 in MCF-7 cells. J. Breast Cancer.

[22] Gao HY, Huo FC, Wang HY, Pei DS (2017). MicroRNA-9 inhibits the gastric cancer cell proliferation by targeting TNFAIP8. Cell Prolif..

[23] He L, Zhang L, Wang M, Wang W (2017). miR-9 functions as a tumor inhibitor of cell proliferation in epithelial ovarian cancer through targeting the SDF-1/CXCR4 pathway. Exp. Ther. Med..

[24] Zhu M, Xu Y, Ge M, Gui Z, Yan F (2015). Regulation of UHRF1 by microRNA-9 modulates colorectal cancer cell proliferation and apoptosis. Cancer Sci..

[25] Wang H (2015). miR-9 promotes cell proliferation and inhibits apoptosis by targeting LASS2 in bladder cancer. Tumour Biol..

[26] Wei N (2016). MicroRNA-9 Mediates the Cell Apoptosis by Targeting Bcl2l11 in Ischemic Stroke. Mol. Neurobiol..

[27] Wang K, Long B, Zhou J, Li PF (2010). miR-9 and NFATc3 regulate myocardin in cardiac hypertrophy. J. Biol. Chem..

[28] Jeyabal P (2016). MicroRNA-9 inhibits hyperglycemia-induced pyroptosis in human ventricular cardiomyocytes by targeting ELAVL1. Biochem. Biophys. Res. Commun..

[29] Bateman NW (2004). Intestinal tumor progression is associated with altered function of KLF5. J. Biol. Chem..

[30] Chen C (2002). A possible tumor suppressor role of the KLF5 transcription factor in human breast cancer. Oncogene.

[31] Chen C (2003). KLF5 is frequently deleted and downregulated but rarely mutated in prostate cancer. Prostate.

[32] Suzuki T (2009). Kruppel-like factor 5 shows proliferation-specific roles in vascular remodeling, direct stimulation of cell growth, and inhibition of apoptosis. J. Biol. Chem..

[33] Ghaleb AM (2005). Kruppel-like factors 4 and 5: the yin and yang regulators of celluar proliferation. Cell Res..

[34] Yang Y (2005). KLF4 and KLF5 regulate proliferation, apoptosis and invasion in esophageal cancer cells. Cancer Biol. Ther..

[35] Shindo T (2002). Kruppel-like zinc-finger transcription factor KLF5/BTEB2 is a target foe angiotensin II signaling and essential regulator of cardiovascular remodeling. Nat. Med..

[36] Sun C (2017). MicroRNA-98 negatively regulates myocardial infarction-induced apoptosis by down-regulating Fas and caspase-3. Sci. Rep..

[37] Cesana M (2011). A long noncoding RNA controls muscle differentiation by functioning as a competing endogenous RNA. Cell.

[38] Liu CY (2018). LncRNA CAIF inhibits autophagy and attenuates myocardial infarction by blocking p53-mediated myocardin transcription. Nat. Commun..

[39] Zhang Y (2018). LncRNA ZFAS1 as a SERCA2a inhibitor to cause intracellular Ca(2+) overload and contractile dysfunction in a mouse model of myocardial infarction. Circ. Res..

[40] Wang K (2016). The long noncoding RNA NRF regulates programmed necrosis and myocardial injury during ischemia and reperfusion by targeting miR-873. Cell Death Differ..

[41] Salmena L, Poliseno L, Tay Y, Kats L, Pandolfi PP (2011). A ceRNA hypothesis: the Rosetta Stone of a hidden RNA language?. Cell.

[42] Xie CH (2016). Long non-coding RNA TUG1 contributes to tumorigenesis of human osteosarcoma by sponging miR-9-5p and regulating POU2F1 expression. Tumour Biol..

[43] Dong JT, Chen C (2009). Essential role of KLF5 transcription factor in cell proliferation and differentiation and its implications for human diseases. Cell Mol. Life Sci..

[44] Diakiw SM, D’Andrea RJ, Brown AL (2013). The double life of KLF5: Opposing roles in regulation of gene-expression, cellular function, and transformation. IUBMB Life.

[45] Shi Q (2016). Kruppel-like factor 5 promotes apoptosis triggered by tumor necrosis factor alpha in LNCaP prostate cancer cells via up-regulation of mitogen-activated protein kinase kinase 7. Urol. Oncol..

[46] Tarapore RS, Yang Y, Katz JP (2013). Restoring KLF5 in esophageal squamous cell cancer cells activates the JNK pathway leading to apoptosis and reduced cell survival. Neoplasia.

[47] Huang W (2016). Combination of microRNA-21 and microRNA-146a attenuates cardiac dysfunction and apoptosis during acute myocardial infarction in mice. Mol. Ther. Nucleic Acids.

