# Supplementary material for: The long non-coding RNA TUG1-miR-9a-5p axis contributes to ischemic injuries by promoting cardiomyocyte apoptosis via targeting KLF5
Source: Cell Death Dis. 2019 Dec 2;10(12):908. doi: 10.1038/s41419-019-2138-4 (PMC6885510; doi:10.1038/s41419-019-2138-4)

**The long non-coding RNA TUG1-miR-9a-5p axis contributes to ischemic injuries by promoting cardiomyocyte apoptosis via targeting KLF5**

Di Yang^1^, Jie Yu^1^, Hui-Bin Liu^1^, Xiu-Qing Yan^1^, Juan Hu^1^, Yang Yu^1^, Jing Guo^1^, Ye Yuan^1^, Zhi-Min Du^1,2,3^*

^1^Institute of Clinical Pharmacy, the Second Affiliated Hospital of Harbin Medical University (The University Key Laboratory of Drug Research, Heilongjiang Province), Harbin 150086, China.

^2^Department of Clinical Pharmarcology（State-Province Key Laboratories of Biomedicine-Pharmaceutics of China, Key Laboratory of Cardiovascular Research, Ministry of Education）, College of Pharmacy, Harbin Medical University, Harbin 150086, China.

^3^State Key Laboratory of Quality Research in Chinese Medicines, Macau University of Science and Technology, Macau, PR China.

*Correspondence:

Zhi-Min Du, Institute of Clinical Pharmacology, the Second Affiliated Hospital of Harbin Medical University, State Key Laboratory of Quality Research in Chinese Medicines, Macau University of Science and Technology, Xuefu Road 246^#^, Nangang District, Harbin 150086, PR China. Tel/Fax:+86-451-86605353;

Email: [dzm1956@126.com](mailto:dzm1956@126.com)

Running title: lncR-TUG1/miR-9/KLF5 regulates apoptosis

**Figure S1**

**
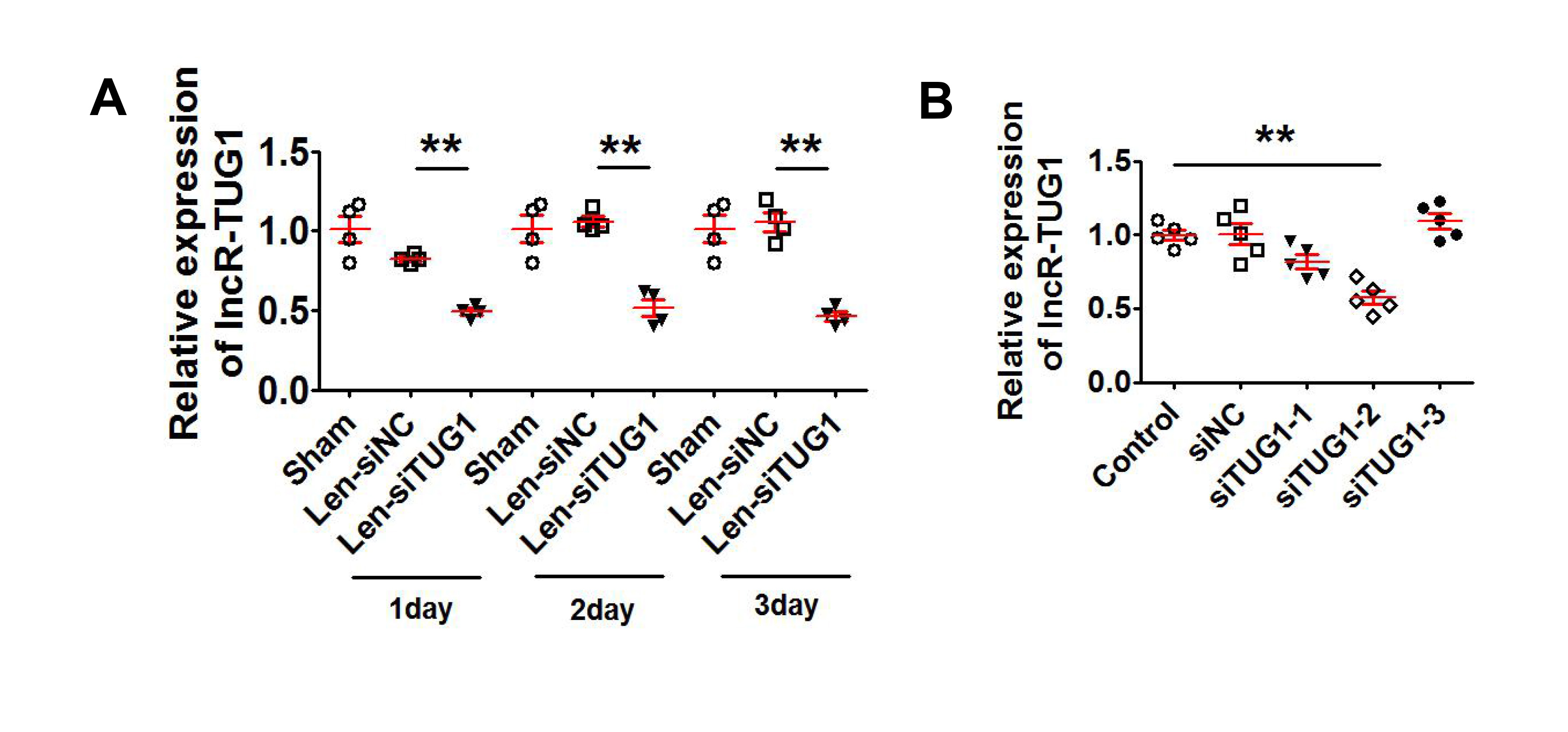
**

**Figure S2**

**
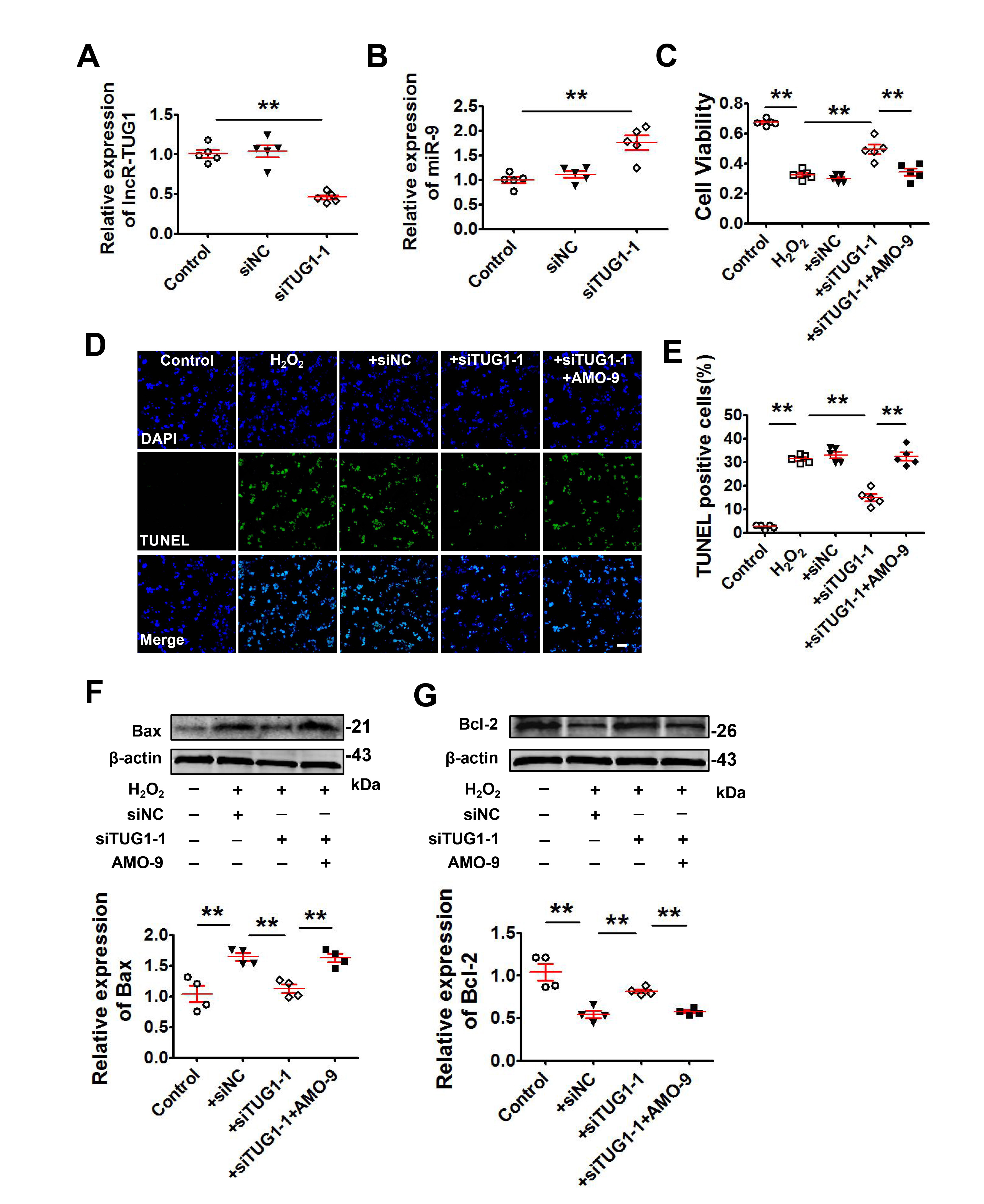
**


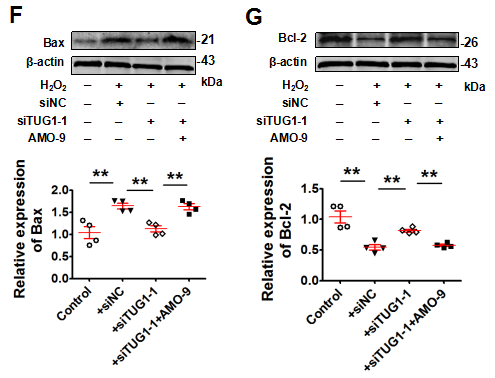


**Figure S3**

**
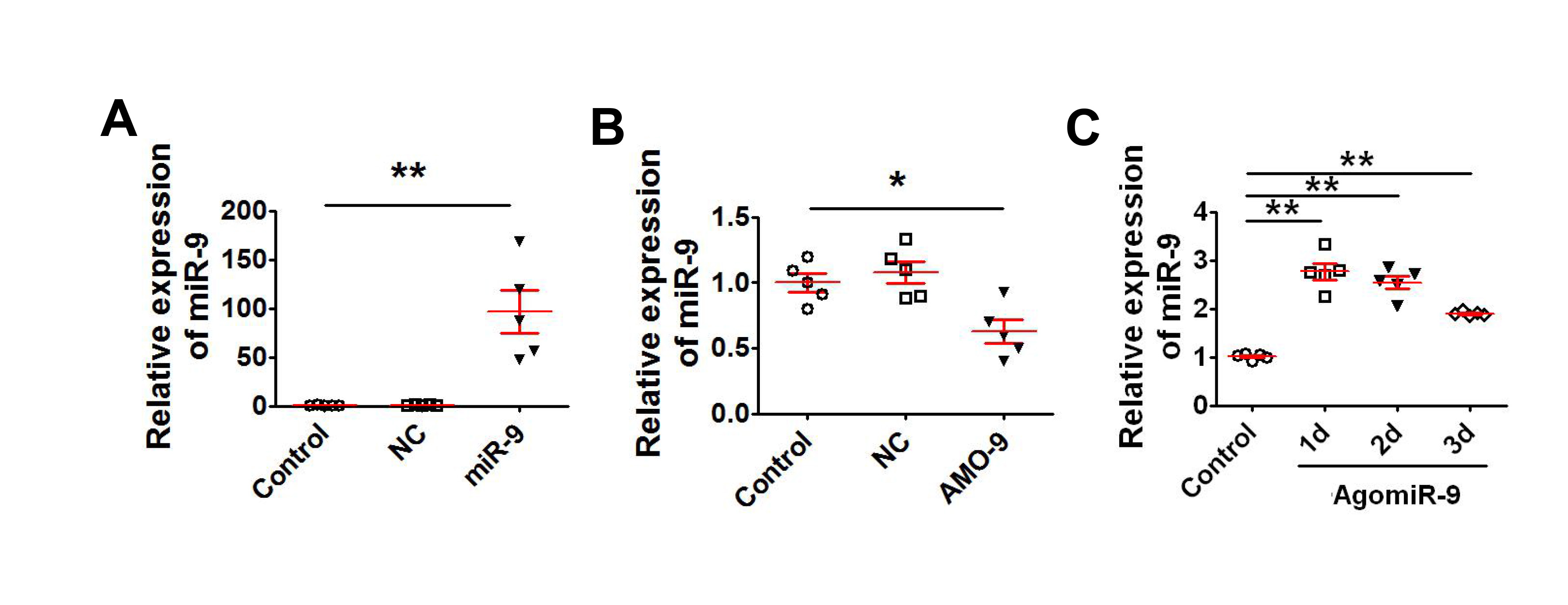
**

**Figure S4**


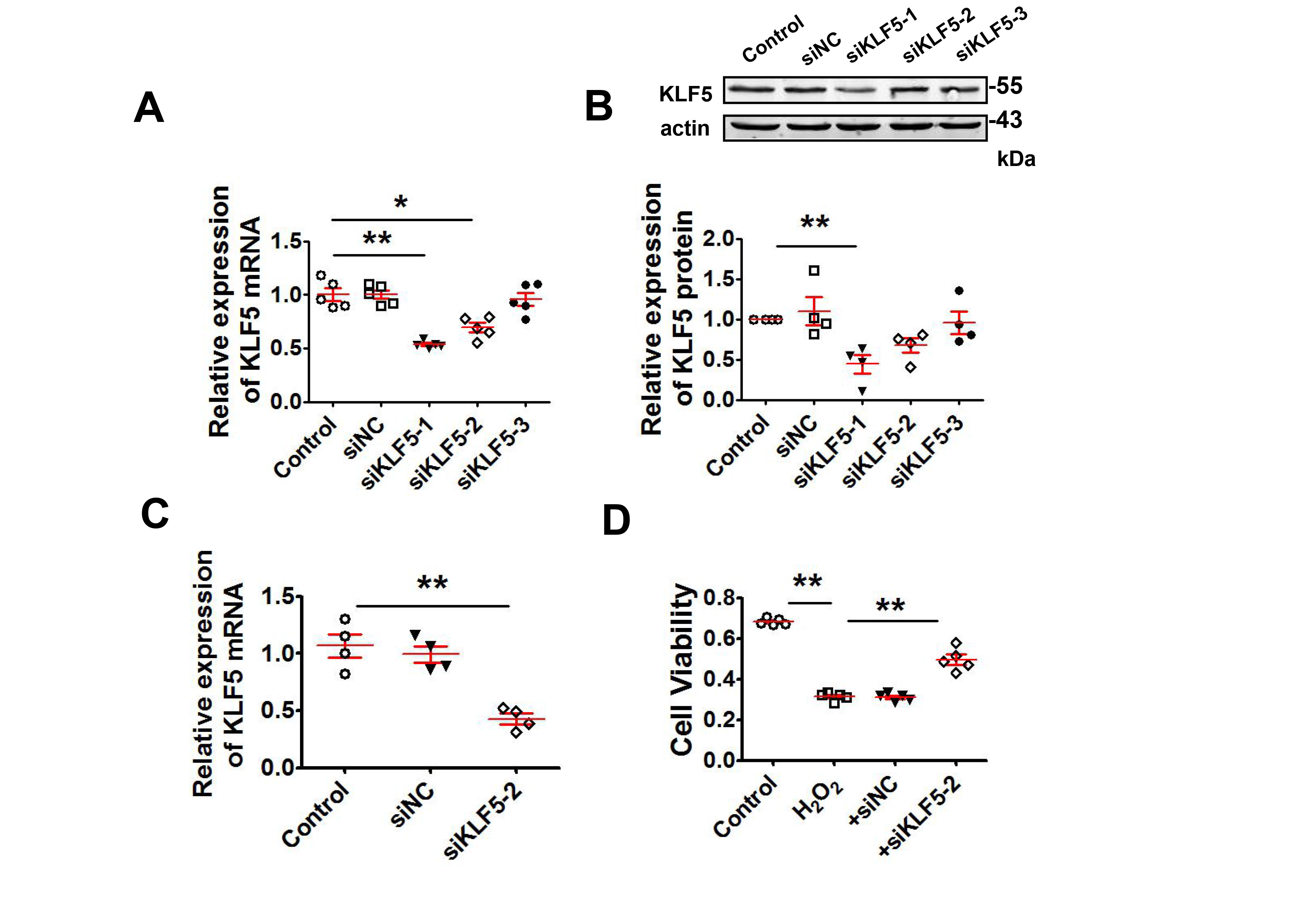

Supplement: Supplementary file 1 — Supplementary Material [file 41419_2019_2138_MOESM1_ESM.doc]
