# Supplementary material for: The long non-coding RNA TUG1-miR-9a-5p axis contributes to ischemic injuries by promoting cardiomyocyte apoptosis via targeting KLF5
Source: Cell Death Dis. 2019 Dec 2;10(12):908. doi: 10.1038/s41419-019-2138-4 (PMC6885510; doi:10.1038/s41419-019-2138-4)
Supplement: Supplementary file 2 — Supplementary figure legends [file 41419_2019_2138_MOESM2_ESM.doc]

**Figure S1. Transfection efficiency of lncR-TUG1 in NRVMs and heart tissue.**

(A) LncR-TUG1 expression level in mice ventricles after administration with the lentiviral vectors carrying siTUG1 (Len-siTUG1) (n = 5). ***P* < 0.01 by Student’s t-test. Data are presented as mean ± SEM. (B) Three siRNAs of lncR-TUG1 (siTUG1-1, siTUG1-2 and siTUG1-3) were designed and lncRNA-TUG1 expression was knockdown by siTUG1-2 in neonatal rat ventricular myocytes (NRVMs). Therefore, siTUG1-2 was chosen for the following experiments. n = 5. ***P*<0.01 by Student’s t-test. Data are presented as mean ± SEM.

**Figure S2. Silencing lncR-TUG1 by transfeced siTUG1-1 alleviates cardiomyocyte apoptosis.**

(A) LncRNA-TUG1 expression was knockdown by siTUG1-1 (C = 100nmol/L) in neonatal rat ventricular myocytes(n = 5). ***P*<0.01 by one-way ANOVA analysis with Tukey's multiple comparison test. Data are presented as mean ± SEM. (B) Increase in miR-9 level after LncR-TUG1 knockdown by siTUG1-1 (n = 5). ***P*< 0.01 by Student’s t-test. Data are presented as mean ± SEM. (C) Alterations of cell viability of NRVMs by MTT assay (n = 5). ***P*<0.01 by one-way ANOVA analysis with Tukey's multiple comparison test. Data are presented as mean ± SEM. (D) Representative images of TUNEL staining of NRVMs for DNA defragmentation showing the apoptotic cells (scale bar: 60 μm). (E) Statistical results of TUNEL-positive cells per field (n = 5). ***P*<0.01 by one-way ANOVA analysis with Tukey's multiple comparison test. Data are presented as mean ± SEM. (F)(G) Western blot analysis of protein levels of Bax (n = 4) and Bcl-2 (n = 4) in NRVMs with different treatments. ***P*<0.01 by one-way ANOVA analysis with Tukey's multiple comparison test. Data are presented as mean ± SEM.

**Figure S3. Transfection efficiency of miR-9 in NRVMs and heart tissue.**

(A) miR-9 expression level in NRVMs after miR-9 transfection (n = 5). (B) miR-9 expression level in NRVMs after AMO-9 transfection (n = 5). **P* < 0.05, ***P* < 0.01 by Student’s t-test. Data are presented as mean ± SEM. (C) miR-9 expression level in mice ventricles after administration with agomiR-9 (n = 5). ***P* < 0.01 by Student’s t-test. Data are presented as mean ± SEM.

**Figure S4. Transfection efficiency of KLF5 in NRVMs.**

Three siRNAs of KLF5 (siKLF5-1, siKLF5-2 and siKLF5-3) were designed and KLF5 expression was silenced in mRNA (A) and protein (B) level by siKLF5-1. n = 4. **P* < 0.05, ***P* < 0.01. Data are presented as mean ± SEM. (C) KLF5 expression was knockdown by siKLF5-2 (C = 100nmol/L) in NRVMs. (D) Alterations of cell viability of NRVMs by MTT assay (n = 6). ***P*<0.01 by one-way ANOVA analysis with Tukey's multiple comparison test. Data are presented as mean ± SEM.
